# Seroprevalence and Seroconversion of Dengue and Implications for Clinical Diagnosis in Amazonian Children

**DOI:** 10.1155/2014/703875

**Published:** 2014-12-08

**Authors:** Antonio Camargo Martins, Thasciany Moraes Pereira, Humberto Oliart-Guzmán, Breno Matos Delfino, Saulo Augusto Silva Mantovani, Athos Muniz Braña, Fernando Luiz Cunha Castelo Branco, José Alcântara Filgueira Júnior, Ana Paula Santos, Alanderson Alves Ramalho, Andréia Silva Guimarães, Thiago Santos de Araújo, Cristieli Sérgio de Menezes Oliveira, Benedito Antônio Lopes da Fonseca, Mônica da Silva-Nunes

**Affiliations:** ^1^Centro de Ciências da Saúde e do Desporto, Universidade Federal do Acre, Campus Universitário, BR 364, Bairro Distrito Industrial, 69.919-900 Rio Branco, AC, Brazil; ^2^Faculdade de Medicina de Ribeirão Preto, Universidade de São Paulo, 14049-900 Ribeirão Preto, SP, Brazil

## Abstract

This study aimed to evaluate the prevalence of serum IgG dengue in children in an Amazonian population, to assess the seroconversion rate in 12 months, and to estimate how many seropositive children had a prior clinical diagnosis of dengue. We conducted a population-based study between 2010 and 2011, with children aged 6 months to 12 years that were living in the urban area of a small town in the Brazilian Amazon. The prevalence of IgG antibodies against dengue antigens was determined by indirect ELISA technique, and seronegative children were reexamined after 12 months to determine seroconversion rates. Results showed seroprevalence of IgG antibodies against dengue type of 2.9%, with no significant association between age, race, and sex. In seropositive children, only 8.4% had received a clinical diagnosis of dengue, and the ratio of clinically diagnosed cases and subclinical cases was 1 : 11. The seroconversion rate between 2010 and 2011 was 1.4% (CI 3.8% to 35.1%). The seroprevalence of dengue in this pediatric population was low, and the vast majority of cases were not clinically detected, suggesting a difficulty in making the clinical diagnosis in children and a high frequency of asymptomatic infections.

## 1. Introduction

Dengue is a leading infectious disease, with 96 million symptomatic cases estimated in 2010 [1]. An important feature of dengue is the four serotypes (DEN-1, DEN-2, DEN-3, and DEN-4) and the recent acknowledgement of a new serotype [2], which may run concurrently in a given region, leading to certain epidemics for a specific serotype. Examples of this occurred in Brazil for serotypes DEN-1 and DEN-4 in 1981, DEN-2 in 1990, and DEN-3 in 2002. The last epidemic included 696,472 reported cases [3–7].

Another important point of the disease is that during epidemics the exposed population develops protection against the circulating serotype but not against the other serotypes, resulting in immunity that is serotype-specific. Due to the changes in the circulation of serotypes over the years, older individuals maintain the immunity they have already acquired and acquire immunity to other serotypes, but every year, newborns are likely to be susceptible to the new circulating serotype [8, 9].

In Brazil, more than 1 million cases were reported in 2010 with an incidence of 530.3 cases per 100,000 inhabitants, corresponding to more than 60% of the cases reported worldwide [10]. According to the Ministry of Health, the number of severe cases (dengue hemorrhagic fever and dengue with complications) reported between 2010 and 2012 was 32,445, with 1,465 deaths during this period [11, 12]. The number of cases by age group for this period was 20,185 in children younger than 1 year, 47,420 in children 1–4 years old, 90,845 in children 5–9 years old, and 140,279 in children 10–14 years old. They accounted for 9% and 8% of the cases in these 3 years [11].

The distribution of dengue cases in Brazil has been heterogeneous [6, 7]. In 2001, 51,309 cases were identified in the northern region, but in 2010, the number of cases almost doubled (98,632 cases). In 2010, the northern region had the second highest incidence of dengue cases in Brazil (621 cases per 100,000 inhabitants) and in 2011 had the highest incidence in the country (752 cases per 100,000 inhabitants). This recent increase in incidence in the northern region may be caused by three main factors: increased migration due to investment in the region and the construction of new roads and trade routes that facilitate the transit of persons, the reintroduction of serotype 4 from Roraima in 2011, and the displacement of a vector from large urban centers to small Amazonian localities [13–15]. In 2011, the state of Acre had the highest incidence of dengue fever in Brazil, with 2,571.7 cases per 100,000 inhabitants. In 2014, almost 100 autochthonous dengue cases have been reported in the Vale do Jurua for the first time, a few months after paving of the BR-364, which connects Rio Branco to Cruzeiro do Sul, was completed [16]. Thus, Acre is expecting new foci of dengue epidemics to occur soon in an area of immunologically naïve subjects, which demonstrates how dengue is again becoming a major problem in the Brazilian Amazon.

Regarding the clinical features of dengue in the pediatric age group, one of the most important problems is the difficulty in diagnosis because the criteria proposed (headache, retroorbital pain, and myalgia) are difficult to measure in young children [17]. Biswas et al. [18] reported that 25% of children with positive serology for dengue tested in Nicaragua did not fit the diagnostic criteria proposed by the World Health Organization (WHO). In addition, several febrile pediatric diseases may be included in the differential diagnosis, contrary to what occurs in most older patients [19], complicating the diagnosis of dengue fever and requiring the completion of clinical diagnosis with complete blood count (CBC) in patients under 15 years [17].

Although detecting clinical cases helps estimate the prevalence of dengue, serological surveys in the general population allow us to identify asymptomatic infections and insights into the circulation of the virus, which help to increase awareness of dengue, particularly in the pediatric age group. We performed a serologic survey of children between 2010 and 2011 in a small town in Acre, in the western Brazilian Amazon to identify the seroprevalence of antibodies against dengue antigens in this specific population's age strata.

## 2. Subjects, Material, and Methods

### 2.1. Study Area

Assis Brasil was founded in 1976 from old established communities in an area with rubber plantations. In 2003, the population was estimated at 3,667 inhabitants, and 38% of the population lived in rural areas. In 2010, the estimated population was 6,075 inhabitants, of whom 61% lived in urban areas [20]. Assis Brasil is located 344 km southwest of White River and borders the municipality of Brasileia in the east, the cities of Iñapari (Peru) and Bolpebra (Bolivia) to the south, and the municipality of Sena Madureira to the north. The climate is equatorial, hot, and humid, a subdivision of the tropical climate. The region has a predominantly rainy season between November and April and a predominantly dry season between May and October. The average annual temperature is 24.5°C, ranging between 20°C and 32°C. The relative humidity is 80–90% throughout the year. The annual rainfall is between 1600 mm and 2750 mm. The native vegetation is open forest with palm trees and tropical rainforest [21].

### 2.2. Study Design and Population

In this population-based study, the study population consisted of children who lived in the urban area of Assis Brasil, in the state of Acre, Brazil, in 2010 and 2011. The study was divided into a prevalence study and a seroconversion study.

The dengue prevalence study consisted of a sample of children aged 6 months to 12 years, living in the urban area of Assis Brasil between January and February 2010. Children were located using records maintained by the Family Health Program health workers. The study was explained to the parent or legal guardian, and informed consent was obtained before the study began. Five hundred twelve children between 6 months and 12 years were identified using health records; 411 (80.2%) agreed to have their blood tested for the presence of antibodies against dengue.

The dengue seroconversion study consisted of 292 seronegative children between 6 months to 12 years identified in the 2010 study among the 411 children tested. These children were retested in 2011, 12 months after the first blood sampling. Data and biological samples were collected in January and February 2011, using questionnaires and venous blood sampling. Information was collected on age, sex, self-reported race, place of birth, and residence time of the child in Assis Brasil. The child's mother or guardian was also asked whether the child had ever received a prior clinical diagnosis of dengue from a health worker or medical doctor. The seroconversion aimed to detect subjects that were previously negative to dengue antibodies and that had an infection with dengue virus any time between 2010 and 2011. It is not possible, though, to exclude recent dengue infections that still did not have detectable IgG by January 2010.

### 2.3. Sample Collection and Detection of Antibodies

We collected between 3 and 5 mL of venous blood in sterile vacuum tubes with a clot activator. The samples were centrifuged, and the serum was aliquoted and stored at −20°C until serology was performed. Serum samples were tested using commercial enzyme-linked immunosorbent assay (ELISA) kits for indirect qualitative detection of immunoglobulin G (IgG) antibodies against serotypes 1, 2, 3, and 4 of dengue antigens (PanBio, Inverness Medical Innovations Australia Pty Ltd., Australia), according to the manufacturer's instructions.

### 2.4. Statistical Analysis

A database was created with SPSS 13.0 software (SPSS Inc., Chicago, IL). The distribution of the independent variables was identified through analysis of variance (ANOVA) to compare the means, and the chi-square or Fisher test was used to compare the frequencies or proportions, with significance established at *α* = 0.05.

The overall seroconversion rate and confidence intervals were calculated between 2010 and 2011, using the binomial exact probability computing Epicalc package for R 2.14.0 (R Foundation for Statistical Computing, Vienna) software. Seroconversion rates for specific age strata were also calculated using the same procedures. Differences were considered statistically significant when the *P* value was less than 0.05, obtained with the Fisher or chi-square test. The positive and negative predictive values of the information reported by the mother or guardian of the child were also calculated as already having had dengue before, considering the gold standard serologic status.

### 2.5. Ethical Aspects

The study is part of a project on Child Health approved by the Ethics Committee for Research in Humans of the Federal University of Acre in 2009 (protocol number 23107.008153/2010-92). Informed consent was obtained from the parent or legal guardian of each participant before the study began.

## 3. Results

Of the 411 children tested in 2010, 211 (51.3%) were male, and 200 (48.7%) were female. The mean age was 5.15 years (median = 3.9 years, minimum 6 months, and maximum 11.8 years). The mean residence time in the city was 4.79 years (median = 3.79, minimum 15 days, and maximum 11.8 years). Regarding place of birth, 397 children were born in Acre, 3 were born in the Peruvian Amazon or Bolivia, 1 was born in Rondônia, and 8 did not list a place of birth. Regarding distribution by race, 22.1% of children were reported to be Caucasian by their parents, 2.8% were reported to be black, 7.5% were reported to be Indian, and 67.6% were reported to be mulatto. Regarding the presence of dengue symptoms, 1.9% of the children had had a previous dengue episode sometime in their life, as reported by their mother or guardian.

The prevalence of IgG antibodies against the dengue serotype antigen in children under 12 years was 2.9% (12 cases) and negative in 97.1% of children tested (399 children). There was no statistically significant difference in the prevalence of positive tests by age group (*P* = 0.711), race (*P* = 0.242), or gender (*P* = 0.249). Of the 12 children with positive serology (Table 1), 11 were born and had lived in Assis Brasil since birth, and only 1 was born in another city in Acre.

There was no statistically significant difference in the prevalence of antibodies against dengue in children who reported having had a clinical diagnosis of dengue fever (2.7%) and those who denied having had dengue in their lifetime (12.5%, *P* = 0.216). However, of the 12 children with positive serology, only 1 reported having had dengue previously (Table 2).

The seroconversion rate was 1.4% (confidence interval 3.8% to 35.1%). Only four children had detectable antibodies in 2011, 2 aged 2 to 3 years and a child older than 5 years.

## 4. Discussion

Over the past 10 years, the number of dengue cases in Acre has increased considerably. In 2004, 4,458 cases were reported, rising to 19,406 cases in 2009 and 34,316 cases in 2010 [12]. The majority of these cases occurred in the state capital. In other cities in Acre, dengue cases increased only after 2010. The municipality of Assis Brasil reported only 1 case of dengue in 2004 and 30 cases in 2009 and did not report any cases between 2005 and 2008.

In 2010, some cities in the interior of the Acre state reported an increased number of reported dengue cases [12]. Assis Brasil had the highest incidence rate (409 cases per 10,000 inhabitants), followed by Acrelandia (287.1 cases/10,000 inhabitants), Brasiléia (175 cases/10,000 inhabitants), Xapurí (161.7 cases/10,000 inhabitants), and Capixaba (152 cases/10,000 inhabitants). With the exception of Acrelandia, these cities are located along the Interoceanic Highway, paved in 2003, which may have promoted the internalization of the disease in the southwestern region of the state. In the other municipalities in Acre, a few cases were reported in 2010, but in geographically isolated municipalities (without road access), no cases were reported in 2010 [12]. Meanwhile, with the recent paving of the BR-364 to Cruzeiro do Sul, almost 100 cases have been reported in 2014 [16], and more are expected in the next few months or year. Of the 249 cases reported in 2010 in Assis Brasil in the National Health Information System, only 5.22% [12] occurred in children between 0 and 10 years.

Between 2009 and 2010, 12 cases of dengue in children between 0 and 10 years living in Assis Brasil were reported to the Ministry of Health. Since in our serological study only 8.3% of the pediatric cases identified through serology were clinically detected (and therefore a ratio of 11 subclinical cases for every case with symptoms), the number of infected children in the county between 2009 and 2010 is likely about ten times higher than that reported to the Ministry of Health. Tien et al. [22] found a rate of 3–6 cases of asymptomatic dengue infection for each case detected clinically in children between 2 and 10 years in Vietnam. Regarding the origin of the cases detected in our study, the majority of the seropositive children likely became infected in Assis Brasil, since 91.6% of the children enrolled in the study were born in this city.

The most important finding in our study is that most children with detectable antibodies against the dengue virus were not diagnosed clinically by a health care professional or their family. Studies conducted in Asia suggest that age is related to the appearance of symptoms of dengue, both in primary and secondary infections, and that the occurrence of asymptomatic dengue in a community in a given year leads to the emergence of serious cases in the following years [23, 24].

The difficulty encountered in diagnosing dengue in children is caused by many factors, such as the large numbers of febrile diseases that affect the pediatric population and the fact that children do not present specific signals for dengue [25, 26]. Therefore, the characterization of dengue in the pediatric population is a very important topic, emphasizing the importance of establishing new diagnostic criteria for this population group, since laboratory tests are not always available for diagnosis.

Population-based studies to determine seroconversion in pediatric patients with dengue are scarce in Brazil and worldwide. Graham et al. [27] reported an incidence rate of up to 7.7% in children 4–9 years old in Indonesia between 1994 and 1995. Thai et al. [28] reported a 17.3% seroincidence rate among Vietnamese preschoolers between 2004 and 2005. Tien et al. [22] found a seroconversion rate between 16.9% and 40.4% in children 2–10 years old in Vietnam. Other studies conducted in Nicaragua and Thailand showed variable rates of seroconversion in children [24, 29]. Teixeira et al. [30] found a seroconversion rate of 33.2% in children 0–3 years old in Salvador, Bahia, and the primary risk factor was age. However, differences in transmission levels and the characteristics of each region make it difficult to compare these data with our study.

The cross-sectional design of the study precludes optimal assessment of seroconversion in the case of recent dengue infections occurring a few days prior to the first blood sampling performed in January-February 2010. In this case, these children would not have detectable IgG antibodies yet by the time they were interviewed. However, even in this case it would indicate recent circulation of the virus, which minimizes the possible classification bias of the study.

## 5. Conclusions

This study shows the circulation of the dengue virus in the more remote areas of the Brazilian Amazon and suggests the difficulty of diagnosing dengue in children. Further studies on the relationship between the dengue virus and the pediatric population are needed to assess the true proportion of asymptomatic infections and cases that present diagnostic difficulties in settings with poor diagnostic resources such as the Amazon.

## Figures and Tables

**Table 1:** Prevalence of IgG antibodies against dengue virus antigens in children between 6 months and 12 years, Assis Brasil, Acre, 2010.

Age strata	Number of subjects tested	Number (%) of positive results
6 months to 11 months	18	0 (0,0%)
1–4 years	256	7 (2,7%)
5–9 years	79	2 (2,5%)
10–12 years	58	3 (5,2%)

Total	411	12 (2,9%)

*P* = 0,71, Fisher's exact test.

**Table 2:** Proportion of cases in children with a clinical diagnosis of dengue according to serological results, Assis Brasil, 2010.

Referring to a prior diagnosis of dengue	Negative serology	Positive serology	Total
No	392 (97,3%)	11 (2,7%)	403
Yes	7 (87,5%)	1 (12,5%)	8

Total	399 (97,1%)	12 (2,9%)	411

*P* = 0,213, Fisher's exact test.
